# Identification of the pyroptosis-related prognostic gene signature and characterization of tumor microenvironment infiltration in triple-negative breast cancer

**DOI:** 10.3389/fgene.2022.929870

**Published:** 2022-08-25

**Authors:** Ji Liu, Jianli Ma, Qingyuan Zhang

**Affiliations:** ^1^ Department of Medical Oncology, Harbin Medical University Cancer Hospital, Harbin Medical University, Harbin, Heilongjiang, China; ^2^ Department of Radiation Oncology, Harbin Medical University Cancer Hospital, Harbin, Heilongjiang, China

**Keywords:** pyroptosis, triple-negative breast cancer, tumor microenvironment, immune infiltration, immunotherapy

## Abstract

**Background:** Triple-negative breast cancer remains a highly malignant disease due to the lack of specific targeted therapy and immunotherapy. A growing body of evidence supports the role of pyroptosis in tumorigenesis and prognosis, but further exploration is needed to improve our understanding of the tumor microenvironment in patients with triple-negative breast cancer.

**Methods:** Consensus clustering analysis was performed to construct pattern clusters. A correlation analysis was conducted between the pattern clusters and the tumor microenvironment using GSVA, ESTIMATE, and CIBERSORT. Then, a risk score and a nomogram were constructed and verified to predict overall survival.

**Results:** Two pyro-clusters and three pyro-gene clusters that differed significantly in terms of prognosis, biological processes, clinical features, and tumor microenvironment were identified. The different clusters corresponded to different immune expression profiles. The constructed risk score predicted patient prognosis and response to immunotherapy. Patients with low risk scores exhibited favorable outcomes with increased immune cell infiltration and expression of immune checkpoint molecules. Compared to other models, the nomogram was extremely effective in predicting prognosis.

**Conclusion:** In the landscape of the immune microenvironment, pyroptosis-mediated pattern clusters differed markedly. Both the developed risk score and the nomogram were effective predictive models. These findings could help develop customized treatment for patients with triple-negative breast cancer.

## Introduction

Triple-negative breast cancer (TNBC) is a highly aggressive malignant tumor with a poor prognosis that lacks expression of estrogen and progesterone receptors, as well as the overexpression of the HER2 protein (Dent et al., 2007). For patients with locally advanced or metastatic TNBC, the clinical outcome is not promising, as these patients do not respond to hormonal therapy or targeted agents (Andre and Zielinski, 2012). TNBC is a highly heterogeneous cancer, and researchers have been developing a variety of molecular subtypes that may contribute to individualized therapy (Garrido-Castro et al., 2019). Therefore, a thorough understanding of the pathogenesis and biological characteristics of TNBC can contribute to establishing effective individualized treatment strategies.

Unlike other forms of programmed cell death, pyroptosis is triggered by inflammation (Fang et al., 2020). Pyroptosis is controlled by pyroptosis-related genes (PRGs) involved in signaling pathways, and changes in PRG expression and function play an important role in pyroptosis (Xia et al., 2019; Hou et al., 2020). Tetraarsenic hexoxide may induce pyroptosis by activating the mitochondrial ROS-mediated GSDME pathway, thus inhibiting tumor growth and metastasis of TNBC cells (An et al., 2021). Cisplatin induces pyroptosis by activating the MEG3/NLRP3/caspase 1/GSDMD pathway in TNBC to exert antitumor effects (Yan et al., 2021).

The composition of the tumor microenvironment (TME) is related to tumorigenesis and progression (Turley et al., 2015; Fridman et al., 2017). Recent studies have confirmed that tumor-infiltrating lymphocytes (TILs) of untreated breast cancer patients can effectively predict response to treatment. TNBC is known as lymphocyte-dominant breast cancer, and infiltration of CD8^+^ T and CD4^+^ T cells can predict a survival benefit in TNBC (Stanton and Disis, 2016). Immunotherapy has shown only modest clinical efficacy in breast cancer, and PD-L1 expression has been shown to be associated with TIL infiltration and better clinical outcomes (Cimino-Mathews et al., 2016). Several published reports have demonstrated that pyroptosis interacts with antitumor immune cells to trigger robust antitumor immunity in the TME (Xi et al., 2019; Wang et al., 2020; Zhang et al., 2020). However, current research is restricted to a few pyroptosis modifiers and lymphocytes, and a thorough examination of the TME infiltration features mediated by pyroptosis could contribute to a better understanding of antitumor immunity.

In this study, we identified different pattern clusters mediated by pyroptosis and found that specific clusters differed significantly in terms of prognosis, biological process, clinical features, and TME. Ultimately, we constructed a risk score and a nomogram that effectively predicted overall survival (OS). These findings could aid in the development of personalized treatment for TNBC patients.

## Materials and methods

### Data sources

**FIGURE 1:**
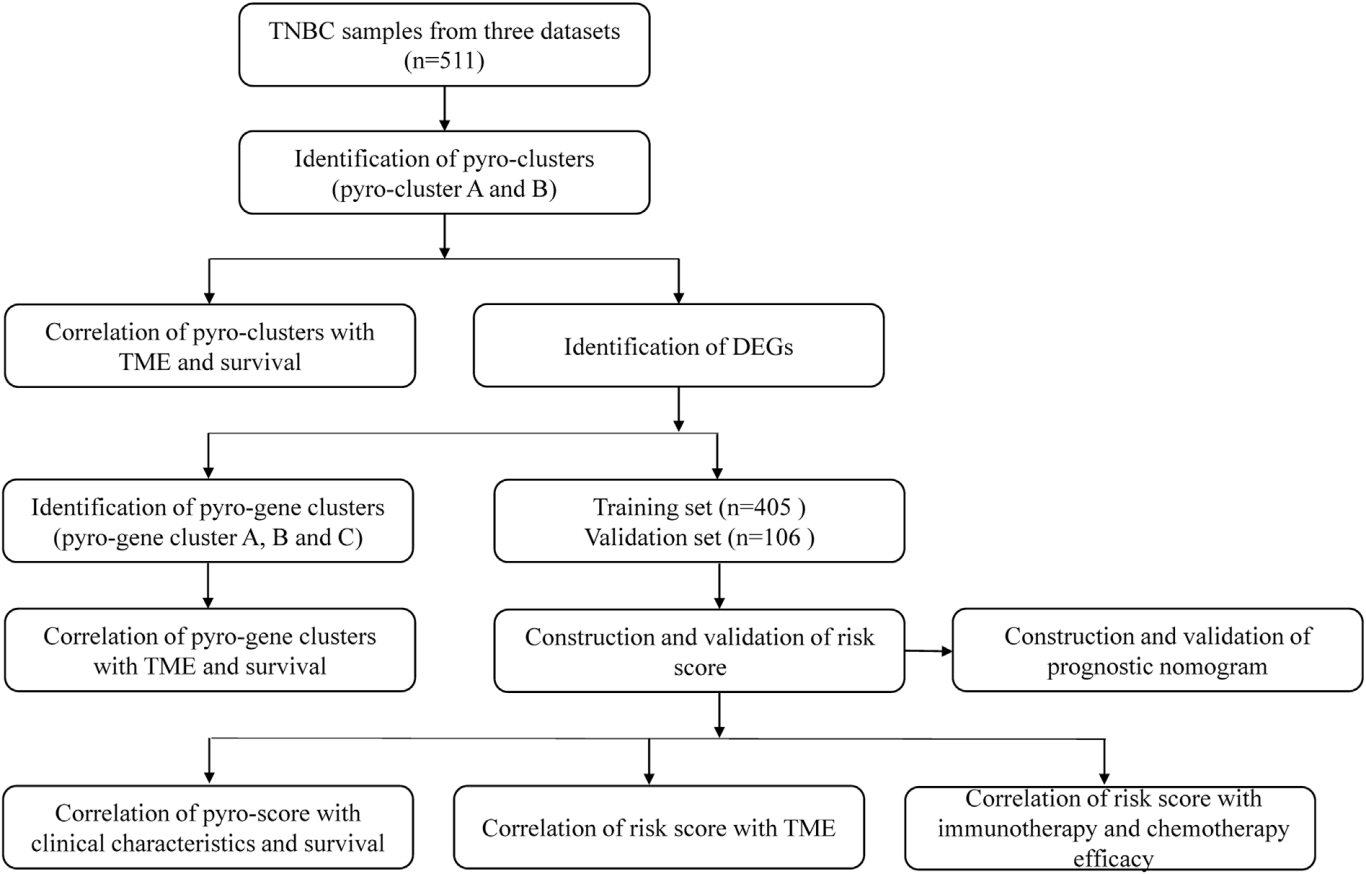
Overview of the analytical process of the study.

Figure 1 shows a map of the process of the present work. The inclusion criteria for datasets were based on the following: 1) datasets with a sufficient sample size greater than 80 were selected; 2) ER, PR, and HER2 status were all negative. The exclusion criteria for datasets were based on the following: 1) patient sample data with an overall survival time of <30 days were excluded; 2) patient samples without clinical characteristics were removed. Ultimately, date of patients with TNBC extracted from TCGA (*n* = 108) (https://portal.gdc.cancer.gov/), METABRIC (*n* = 318) (http://www.cbioportal.org/), and GSE58812 (*n* = 106) (https://www.ncbi.nlm.nih.gov/geo/) were included in the study. The clinicopathological information of these 511 patients is presented in Supplementary Table S2. Forty-four pyroptosis-related genes (PRGs) were retrieved from the Molecular Signatures Database (https://www.gsea-msigdb.org/) and presented in Supplementary Table S1.

### Pyroptosis-based consensus clustering analysis

Based on the expression of PRGs, a consensus unsupervised cluster analysis was performed to ensure the stable classification of patients into different clusters (Wilkerson and Hayes, 2010). A correlation analysis was conducted between pattern clusters and TME using GSVA, ESTIMATE (Yoshihara et al., 2013), and CIBERSORT (Newman et al., 2015).

### Construction of the risk score

TCGA and METABRIC datasets were used as training sets, and the GSE 58812 dataset was used as a validation set. Differentially expressed genes (DEGs) identified from distinct clusters were subjected to univariate, multivariate, and stepwise regression analysis. To define the risk score, we used the formula risk score = Σ (Expi * coefi).

### Cell culture and quantitative real-time PCR

Breast cancer cell MDA-MB-231 was obtained from the America Type Culture Collection (ATCC, Manassas, VA, United States) and cultured in Dulbecco's modified eagle’s medium (DMEM) with 10% fetal bovine serum (FBS) (HyClone, Logan, UT, United States). The cells were maintained at 37 °C with 5% CO2 in an incubator. Total RNA was extracted from cells by using the TRIzol reagent (Invitrogen) and was used to synthesize cDNA by using the Quantscript RT Kit (Promega). Real-time PCR was performed using SYBR Green (BioRad) on the CFX96 system (BioRad Laboratories, Hercules, CA, United States). β-actin was exploited as an internal reference. The 2–ΔΔCt method was used to calculate mRNA expression. The primer sequences used for analysis are listed in Supplementary Table S3.

### Construction and validation of the nomogram

Clinicopathological characteristics related to OS and risk score were used to construct a nomogram in the training set (Iasonos et al., 2008). The predictive power of the nomogram was also validated in the TCGA and METABRIC sets.

### Statistical analyses

All statistical analyses were performed using R (version 4.1.3). Statistical significance was established at *p* < 0.05.

## Results

### The landscape of genetic and transcriptional alterations of PRGs in TNBC

**FIGURE 2:**
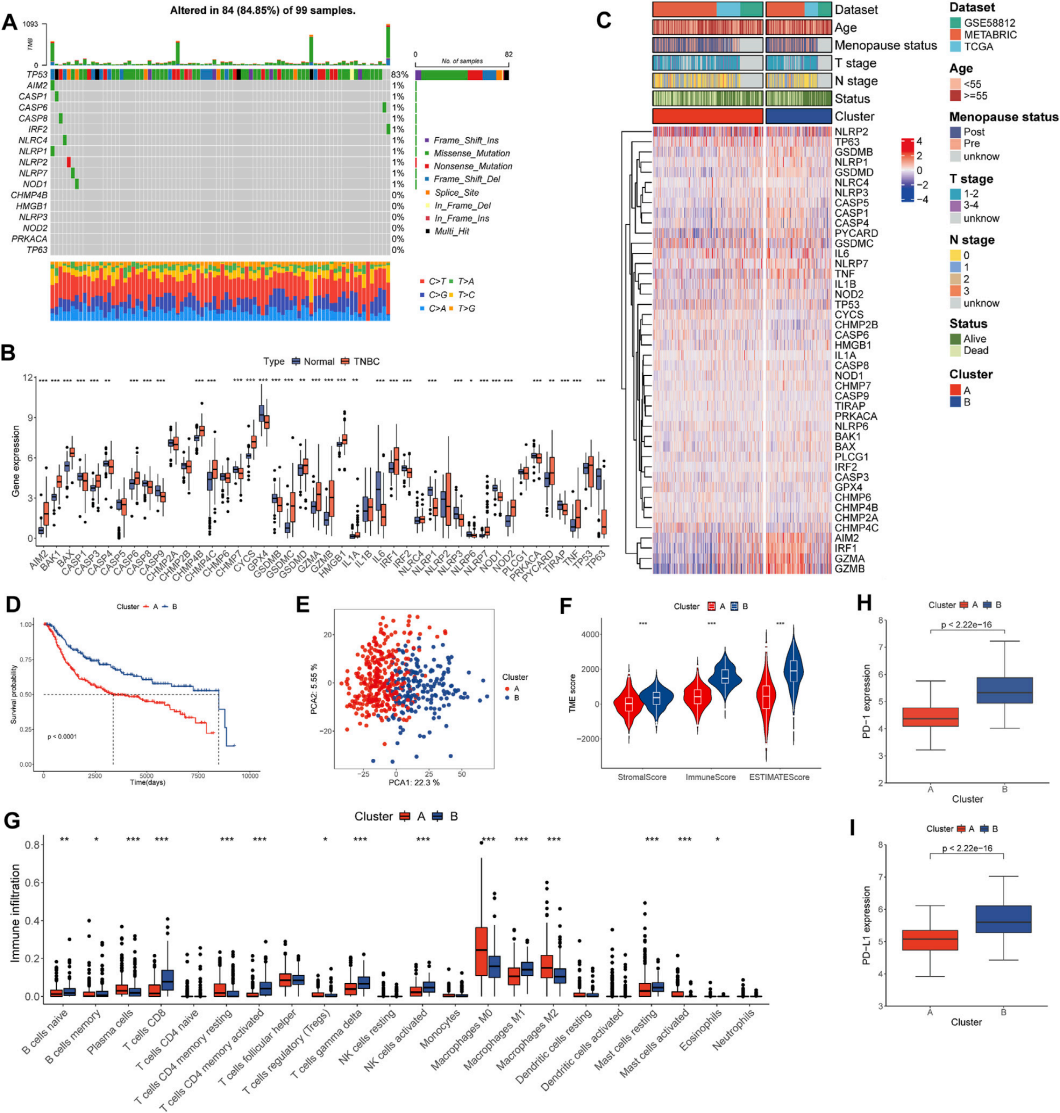
Landscape of genetic and transcriptional alterations and pyro-clusters in patients with TNBC. **(A)** Genetic alteration of PRGs in 99 patients from TCGA cohort. **(B)** Distribution of 44 PRG expression in normal and TNBC tissues. **(C)** Heat map of clinicopathologic features and expression of PRGs between two distinct clusters. **(D)** Kaplan–Meier curves of OS for TNBC patients of the two distinct clusters. **(E)** PCA analysis showing the marked difference in transcriptomes across pyro-clusters. **(F)** Correlations between pyro-clusters and the TME score. **(G)** Abundance of 22 infiltrating immune cell types in the two pyro-clusters. **(H)** Expression \ of PD-1 in the two pyro-clusters. **(I)** Expression of PD-L1 in the two pyro-clusters. *p < 0.05, **p < 0.01, ***p < 0.001.

The prevalence of PRG gene alterations was investigated in the TCGA-TNBC cohort. As shown in Figure 2A, somatic PRG mutations occurred in 84 of the 99 samples, with a mutation frequency of 84.85%. The most mutated gene was TP53 (approximately 83%), followed by AIM2, and CASP8 (approximately 1%). Figure 2B indicated that most genes were differentially expressed in TNBC. We performed prognostic analysis for each PRG and the OS-related PRGs are shown in Supplementary Figure S1. The expression of GZMA, AIM2, NLRP1, NLRP3, NLRP7, GZMB, TNF, IRF1, CASP1, NOD2, IL1B, and CASP4 was linked to better OS.

### Identification of pyro-clusters in TNBC

**FIGURE 3:**
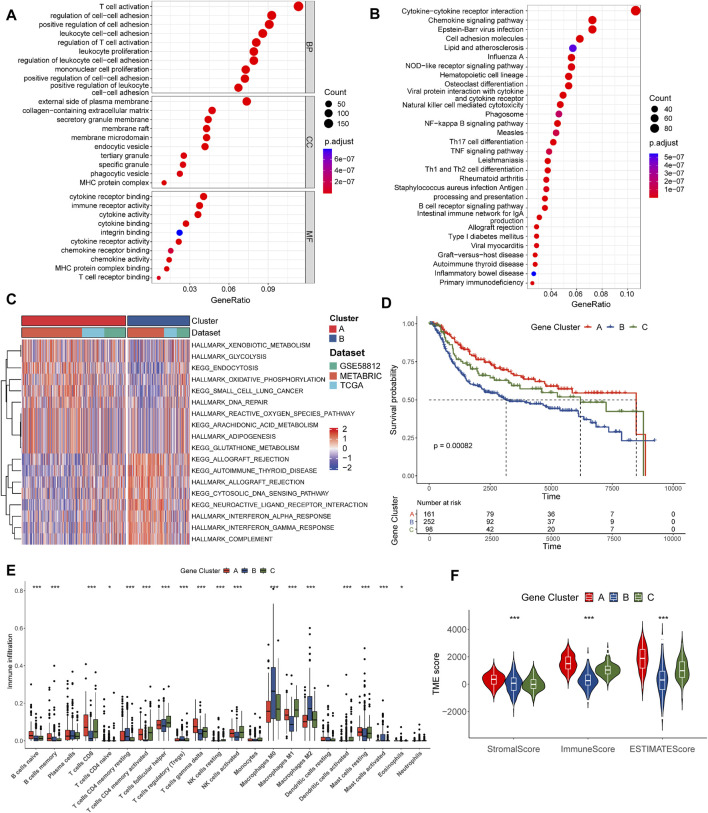
Identification of pyro-gene clusters based on DEGs. **(A)** GO enrichment analyses of DEGs between the two pyro-clusters. **(B)** KEGG enrichment analyses of DEGs between the two pyro-clusters. **(C)** GSVA of biological pathways between two pyro-clusters, in which red and blue represent activated and blue inhibited pathways, respectively. **(D)** Kaplan–Meier curves of OS for TNBC patients between three distinct pyro-gene clusters. **(E)** Abundance of 22 infiltrating immune cell types in the three pyro-gene clusters. **(F)** Correlations between pyro-gene clusters and the TME score. *p < 0.05, **p < 0.01, ***p < 0.001.

Data sets TCGA-TNBC, GSE 58812, and METABRIC-TNBC were combined into a single study cohort. TNBC patients were assigned to two distinct pyroptosis-mediated pattern clusters based on 44 PRG (Supplementary Figure S2). These were designated as pyro-cluster A and pyro-cluster B, with 320 and 191 patients, respectively. PCA analysis revealed that the two clusters could be significantly clustered according to the pyroptotic transcriptional profile (Figure 2E). We subsequently performed survival analysis and the KM curves showed patients in pyro-cluster B had a longer OS (Figure 2D). Furthermore, the association of mRNA profiles of different pyro-clusters with clinicopathological characteristics is shown in Figure 2C. The GSVA results indicated that pathways relevant to immunology and inflamed processes were considerably enriched in pyro-cluster B, such as interferon alpha, interferon-gamma, and the complement (Figure 3C; Supplementary Table S4).

### Correlations of pyro-clusters with the TME in TNBC

The immune score of the patients of pyro-cluster B was higher than that of pyro-cluster A, implying that the TME of the pyro-cluster B had higher immunocyte components (Figure 2F). There were differences in 15 of the 22 lymphocyte subsets in all pyro-clusters, according to the CIBERSORT analysis (Figure 2G). PD-1 and PD-L1 expression was also found to be greater in pyro-cluster B (Figures 2H,I).

### Identification of pyro-gene clusters and exploration of their correlations with the TME

A total of 1778 overlapping DEGs were identified between the two clusters. The results of the functional enrichment analysis are shown in Figures 3A,B; Supplementary Tables S5, 6. Immunology and inflammation pathways were found to be substantially enriched, implying the importance of pyroptosis in the TME.

We further conducted an unsupervised consensus clustering analysis to identify three gene clusters based on DEGs: pyro-gene clusters A, B, and C, comprising 161, 252, and 98 samples, respectively (Supplementary Figure S3). Each pyro-gene cluster produced dramatically different clinical outcomes, according to prognostic analyses (Figure 3D). As shown in Figure 3E, 18 of 22 types of immune cells had notable differences in infiltration. Pyro-gene cluster A had greater infiltration of anti-cancer lymphocytes compared to clusters B and C. The same result was observed in Figure 3F, where the scores of pyro-gene cluster A patients were higher than those of clusters B and C. The above findings revealed separate immune infiltration features among pyro-gene clusters, with pyro-gene cluster A being an immune-inflamed phenotype, cluster B being an immune-desert phenotype, and cluster C being an immune-excluded phenotype.

### Construction and validation of the risk score

To measure the degree of pyroptosis-mediated patterns in each patient, a risk score was constructed. A total of 603 OS-related genes were filtered using univariate Cox regression analysis including the DEG. Following the LASSO regression analysis, 13 genes remained as candidate genes (Supplementary Figure S4). Next, we used multivariate Cox regression analysis to obtain a list of nine genes. The following algorithm was devised using nine genes coefficients:

Risk score = (−0.3233977* expression of CD109) + (0.2387019*expression of DPCD) + (−0.1973012*expression of GTSF1) + (−0.3239561*expression of KLRC3) + (0.1398530*expression of P4HA1) + (−0.2835991*expression of PNMAL1) + (0.1631214*expression of SPRED2) + (−0.1820728 *expression of STAMBPL1) + (−0.2394826*expression of TMEM176A).

**FIGURE 4:**
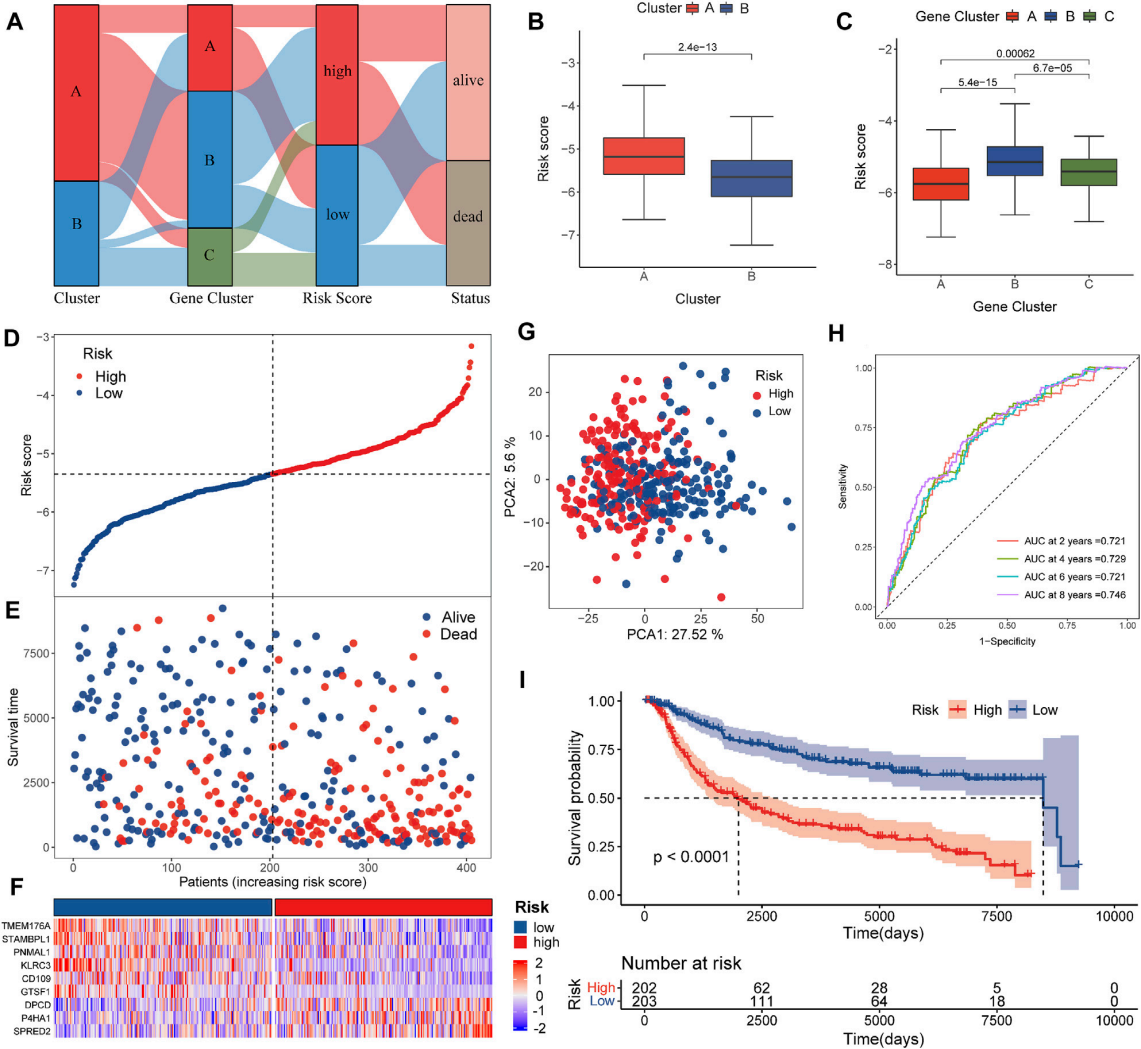
Construction of the risk score in the training set. **(A)** Alluvial diagram of cluster distributions in groups with different risk scores and survival statuses. **(B)** Differences in risk score between the pyro-clusters. **(C)** Differences in risk score between the pyro-gene clusters. **(D,E)** Ranked dot and scatter plots showing the risk score distribution and patient survival status. **(F)** Differences in the expression of nine genes of the prognostic signature between the two pyro-clusters. **(G)** PCA analysis shows a remarkable difference in transcriptomes between the risk groups. **(H)** ROC curves to predict the sensitivity and specificity of 2-, 4-, 6-, and 8-year survival according to the risk score. **(I)** Kaplan–Meier analysis of the OS between the risk groups.

The TNBC patients were divided into various risk groups based on their median values in the training set. Figure 4A revealed the distribution and interaction in the two pyro-clusters, three pyro-gene clusters, two risk score groups, and survival status. Figures 4B,C illustrated the distribution of the risk scores in the two pyro-clusters and three pyro-gene clusters, demonstrating that risk scores may be linked to the characteristics of immune infiltration. High-risk groups were more likely to have worse clinical outcomes than low-risk groups, according to the ranked dot and scatter plots (Figures 4D,E). Figure 4F showed the differential expression of nine genes among risk groups. The expression of nine genes varied dramatically between risk groups, as well as between normal groups (Supplementary Figure S5). Two risk groups exhibited discrete aspects, according to the PCA analysis (Figure 4G).

In the training group, the AUCs of the 2-, 4-, 6-, and 8-year ROC were 0.721, 0.729, 0.721, and 0.746, respectively, showing good predictive ability (Figure 4H). Patients of the validation dataset and of the entire dataset were also separated into two risk sets based on the formula and cut-off values implemented in the training set (Supplementary Figures S6–8). The PCA analysis and scatter plots are shown in Supplementary Figures S6–8, indicating that each patient could also be significantly clustered and their outcome predicted. For all patients in the combined dataset, Supplementary Figures S6–8 confirmed that survival differences were evident across the risk sets. AUCs of the 2-, 4-, 6-, and 8-year ROC (Supplementary Figures S6–8) indicated that the risk score exhibited excellent predictive abilities in the validation and the combined datasets as well.

### Clinical correlation analysis of the risk score

Univariate and multivariate analyses were applied to incorporate OS with the risk score, and clinicopathological characteristics including age, T stage, menopause status, histopathological type, chemotherapy, and N stage. N stage and risk score were potential predictive indicators.

As shown in Supplementary Figure S9, a stratified analysis to evaluate the prediction performance of the risk score in divergent clinical subsets revealed differences in OS for age (*p* < 0.0001), T1-2 (*p* < 0.0001), T3-4 (*p* = 0.1), N0 and N1 (*p* < 0.0001), and N2-3 (*p* = 0.019).

### Correlations of the risk score with the TME in the TNBC

**FIGURE 5:**
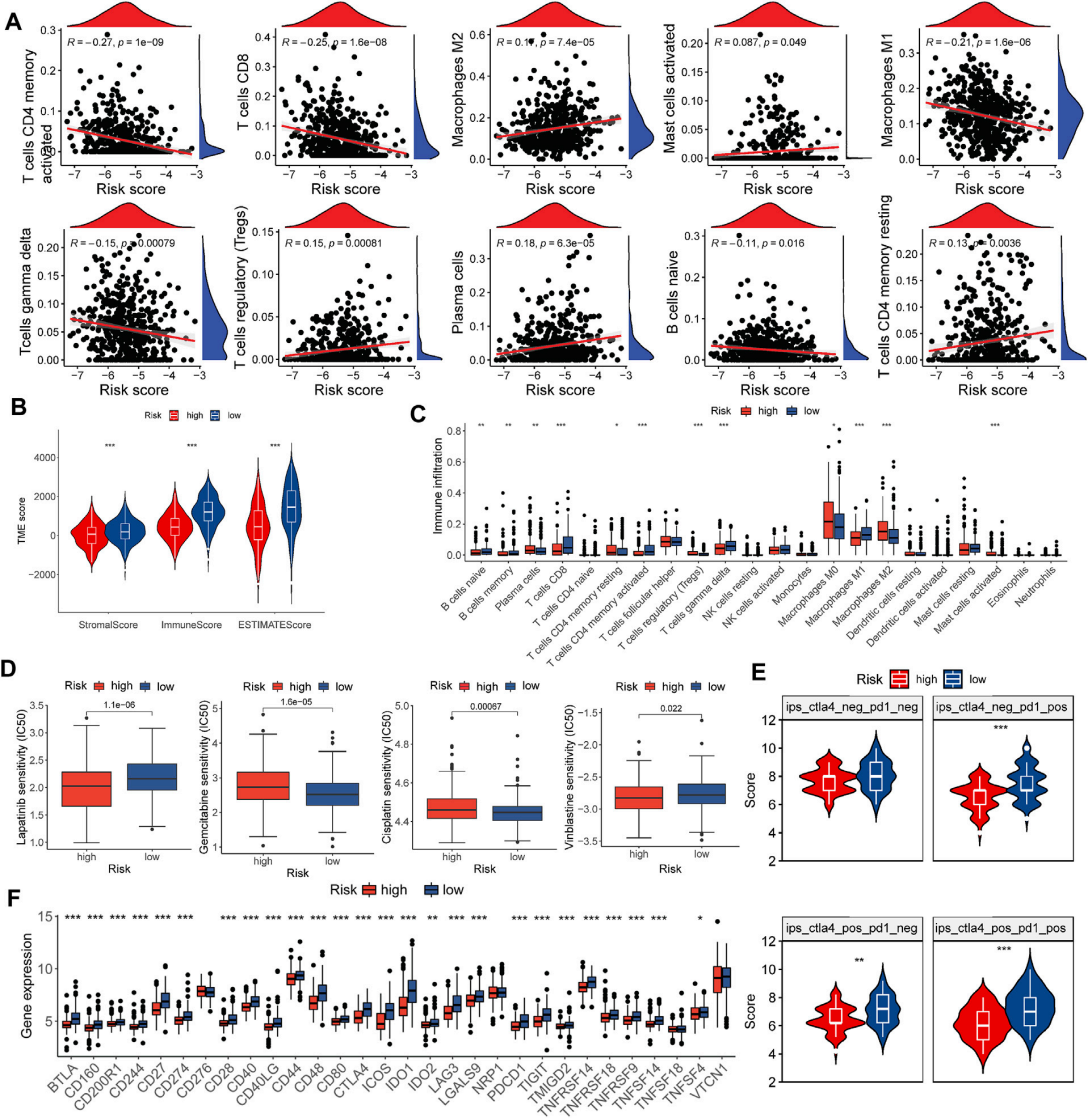
Evaluation of the TME and estimation of the role of the risk score in immunotherapy and chemotherapy efficacy. **(A)** Correlations between the risk score and immune cell types. **(B)** Correlations between the risk score and the TME score. **(C)** Abundance of 22 infiltrating immune cell types in the two risk groups. **(D)** Relationship between the risk score and chemotherapeutic sensitivity. **(E)** Relative distribution of immunotherapy efficacy in the high-risk group versus the low-risk group. **(F)** Expression of immune checkpoints in the high and low-risk groups. *p < 0.05, **p < 0.01, ***p < 0.001.

M2 macrophages, activated mast cells, Tregs, plasma cells, resting CD4 memory T cells, and resting mast cells were considerably and favorably correlated with the risk score, while gamma delta T cells, activated memory CD4 + T cells, CD8 + T cells, M1 macrophages, and naive B cells were unfavorably correlated (Figure 5A). In addition, a low risk score was associated with higher stromal, immune, and estimation scores (Figure 5B), which matched the findings in Figures 4B,C. Among the risk groups, there were also substantial differences in the infiltration of 12 of 22 immune cell types (Figure 5C).

### Estimation of the role of risk score in the efficacy of chemotherapy and immunotherapy

We evaluated several drugs widely used in TNBC treatment across different risk groups by comparing IC50 values and found notable differences between risk groups for lapatinib, vinorelbine, cisplatin, and gemcitabine (Figure 5D).

A recent study demonstrated the ability of IPS to predict the effectiveness of immunotherapy (Charoentong et al., 2017). We assessed differences in risk groups receiving different treatments using the IPS retrieved from the TCIA. In line with our expectations, patients in the low-risk group performed better, which supported our hypothesis that the risk score could be valuable in evaluating the effectiveness of immunotherapy (Figure 5E).

In anti-cancer immunotherapy, immune checkpoint blockers targeting PD-1/CTLA-4 have made a significant contribution, and these immune checkpoints are currently the most widely acknowledged biomarkers for predicting treatment response (Doroshow et al., 2021). Figure 5F shows that 26 of 30 molecules were markedly elevated in the low-risk group. Together, these observations revealed a correlation between the risk score and chemotherapy and immunotherapy efficacy.

### Development of a nomogram to predict OS

**FIGURE 6:**
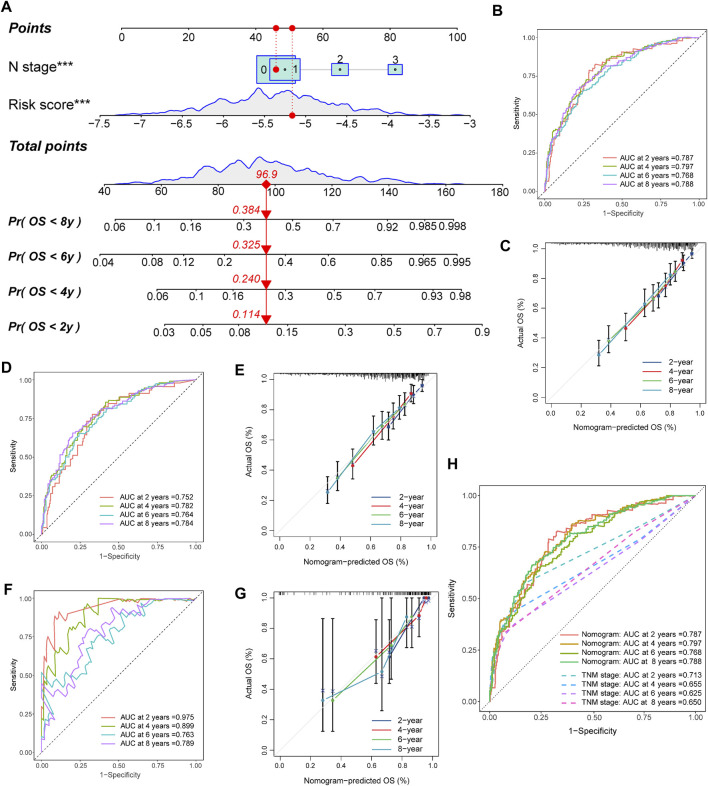
Construction and validation of the nomogram. **(A)** Nomogram for predicting the 2-, 4-, 6-, and 8-year OS of TNBC patients. **(B,C)** Nomogram and calibration curves of the nomogram for predicting the 2-, 4-, 6-, and 8-year OS of TNBC patients in the training set. **(D,E)** Nomogram and calibration curves of the nomogram for predicting the 2-, 4-, 6-, and 8-year OS of TNBC patients in the METABRIC set. **(F,G)** Nomogram and calibration curves of the nomogram for predicting the 2-, 4-, 6-, and 8-year OS of TNBC patients in the TCGA set. **(H)** AUCs of the nomogram and TNM stage for predicting the 2-, 4-, 6-, and 8-year OS of TNBC patients.

We created a nomogram that combines core risk factors and clinical features to predict 2-, 4-, 6-, and 8-year OS rates to simplify the practical application of the risk score. The risk score, age, menopausal status, T stage, and N stage were analyzed as candidate predictors by Cox regression analysis, and the risk score and N stage were ultimately considered the ultimate prognostic elements in the nomogram (Figure 6A). In both the training and external validation sets, the calibration chart, as well as the AUC values for OS at 2, 4, 6, and 8 years, provided evidence supporting the significant discriminatory power of the nomogram (Figures 6B–G). Furthermore, we also compared the predictive accuracy of the nomogram with TNM staging of prognosis, and the nomogram showed better predictive power (Figure 6H). These findings demonstrated that the nomogram had a remarkable capacity to predict survival in patients with TNBC.

### The mRNA relative expression of nine genes in cells

We compared the mRNA levels of nine genes in breast cell line MDA-MB-231 by qRT-PCR analysis. The results showed no detectable expression of TMEM176A and high relative expression of P4HA1, CD109, and STAMBPL1, which may require us to expand the sample size for subsequent functional studies (Supplementary Figure S10).

## Discussion

In this study, based on PRG expression patterns, patients with TNBC were clustered into two distinct pyro-clusters and significant differences were found between the two groups in terms of prognosis and TME characteristics. Pyro-cluster B was remarkably rich in antitumor lymphocyte cell subpopulations, while pyro-cluster A was rich in plasma cells. DEGs identified among pyro-clusters were significantly enriched in immune and tumor-related pathways, demonstrating that the DEGs produced a gene signature leading to different groups mediated by pyroptosis. Based on the DEGs, TNBC patients were assigned to three different pyro-gene clusters. Similarly, alternations in prognostic and TME characteristics between clusters were observed, which were consistent with the results of pyro-clusters. Thus, we constructed a risk score based on DEGs and detected their predictive capacity. Risk scores were distributed according to the different gene expression clusters. Additionally, different risk groups showed significant differences in survival and TME characteristics. Subsequent investigation revealed a substantial correlation between the risk score and immune checkpoint molecules, chemotherapy, and immunotherapy, indicating that the risk score might be an indicator of treatment efficacy. Finally, we constructed a nomogram based on clinicopathological characteristics and risk scores that may be used in clinical practice to predict individual prognosis. We hypothesized that pyroptosis patterns can be used in medical management to detect immunological patterns and guide treatment interventions.

The TME is composed of stromal cells, innate and adaptive immune cells, fibroblasts, and extra-endothelial cells and plays a critical role in tumor progression (Hinshaw and Shevde, 2019). Several reports have indicated that the type and relative content of antitumor lymphocytes are associated with the clinical outcomes of various types of tumors (Dieu-Nosjean et al., 2008; Pages et al., 2009; Denkert et al., 2010; Hwang et al., 2012). Breast cancer presents extensive immunological features, and the phenotype and magnitude of TILs vary between subtypes, with TNBC being the subtype most associated with lymphocyte-predominant breast cancer (Stanton et al., 2016; Denkert et al., 2018). Although CD8^+^ TILs are associated with a better prognosis in TNBC, they show no correlation in patients with hormone receptor-positive tumors (Stanton and Disis, 2016). Natural killer (NK) cells are important effectors of anti-cancer immunity, and their specificity may play an important role in immunotherapy (Imai et al., 2000; Nair and Dhodapkar, 2017). Differences in the infiltration of CD8+T and NK cells were found between the two groups, which were related to OS, a finding in agreement with previous studies. As immunosuppressive cells, increased Treg cell infiltration is associated with unfavorable outcomes and advanced-stage of breast cancer (Plitas et al., 2016). Tumor-associated macrophages (TAMs) are a double-edged sword and interact with TME in the development of breast cancer (Tang, 2013; Yang and Zhang, 2017). TAMs tend to display an M2-like macrophage phenotype, and the abundance of M2-TAMs in breast tumors is correlated with a poor outcome (Wu et al., 2020). Our study came to the same conclusion that pyro-cluster A with a high abundance of Tregs and M2 macrophages exhibited poorer survival, implying their deleterious impact on TNBC progression. These observations suggested that PRGs were intimately linked to the modulation of TME in patients with TNBC.

Immunotherapy is rapidly developing with the advancement of research on tumor immunology. Immune checkpoint inhibitors (ICIs) targeting CTLA-4, PD-1, and PD-L1 have been used in the treatment of other solid tumors, but have shown little success in breast cancer. Several studies have shown partial clinical responses to ICIs in TNBC, including some complete responders (Nanda et al., 2016). Therefore, it is challenging to screen patients who can benefit from immunotherapy. In this study, two pyro-clusters and three pyro-gene clusters with different immunological profiles were identified. We considered that pyro-gene cluster A was immune-inflamed and corresponded to the lowest risk score, pyro-gene cluster B was an immune-desert phenotype, and pyro-gene cluster C was an immune-excluded phenotype, corresponding to the highest risk score. Further analysis confirmed that a low risk score correlated with the expression of immune checkpoint molecules and the response to immunotherapy. Pyroptosis is an inflammatory and immunogenic response that activates TILs to eliminate cancer cells, as well as resist antitumor immunity (Zhang et al., 2020). Gasdermins are currently thought to be mediators of pyroptosis, and their expression enhances the effects of TAMs and TILs. The above findings confirmed the importance of pyroptosis in the TME, as well as the prediction of sensitivity to immunotherapy. The constructed risk score could assess the heterogeneity of pyroptosis patterns and differences in the characteristics of the TME between individuals. Therefore, the risk score achieved a dual predictive power in terms of survival and sensitivity to treatment.

Although the findings of this study present good clinical utility, they still presented certain limitations. Our analysis of the TME was based on mRNA expression, and there may be a genetic overlap between different immune cells, nonetheless, our results were still consistent with previous studies. Prospective data and basic experiments for theoretical verification are still needed in the future to confirm our findings. Furthermore, many important clinical data were not available for further analysis in the dataset, which may have affected the accuracy of our model.

Breast cancer is markedly heterogeneous, while the TNBC phenotype has a unique immunobiological profile compared to other subtypes. Previous studies have focused on identifying the signature of pyroptosis-related genes in breast cancer, and as far as we know, this was the first study to specifically focus on TNBC and establish a prognostic prediction model.

## Conclusion

This study suggests that pyroptosis plays a multifaceted role in TNBC. Pyroptosis-mediated pattern clusters may partially explain the heterogeneity of TNBC. Determining the risk score of a tumor for individual patients can help predict the prognosis and effectiveness of immunotherapy. These findings could aid in the development of customized treatment for patients with TNBC.

## Data Availability

The original contributions presented in the study are included in the article/Supplementary Material; further inquiries can be directed to the corresponding author.
