# Supplementary material for: Identification of the pyroptosis-related prognostic gene signature and characterization of tumor microenvironment infiltration in triple-negative breast cancer
Source: Front Genet. 2022 Aug 25;13:929870. doi: 10.3389/fgene.2022.929870 (PMC9453819; doi:10.3389/fgene.2022.929870)
Supplement: Supplementary file 2 [file Table1.DOCX]

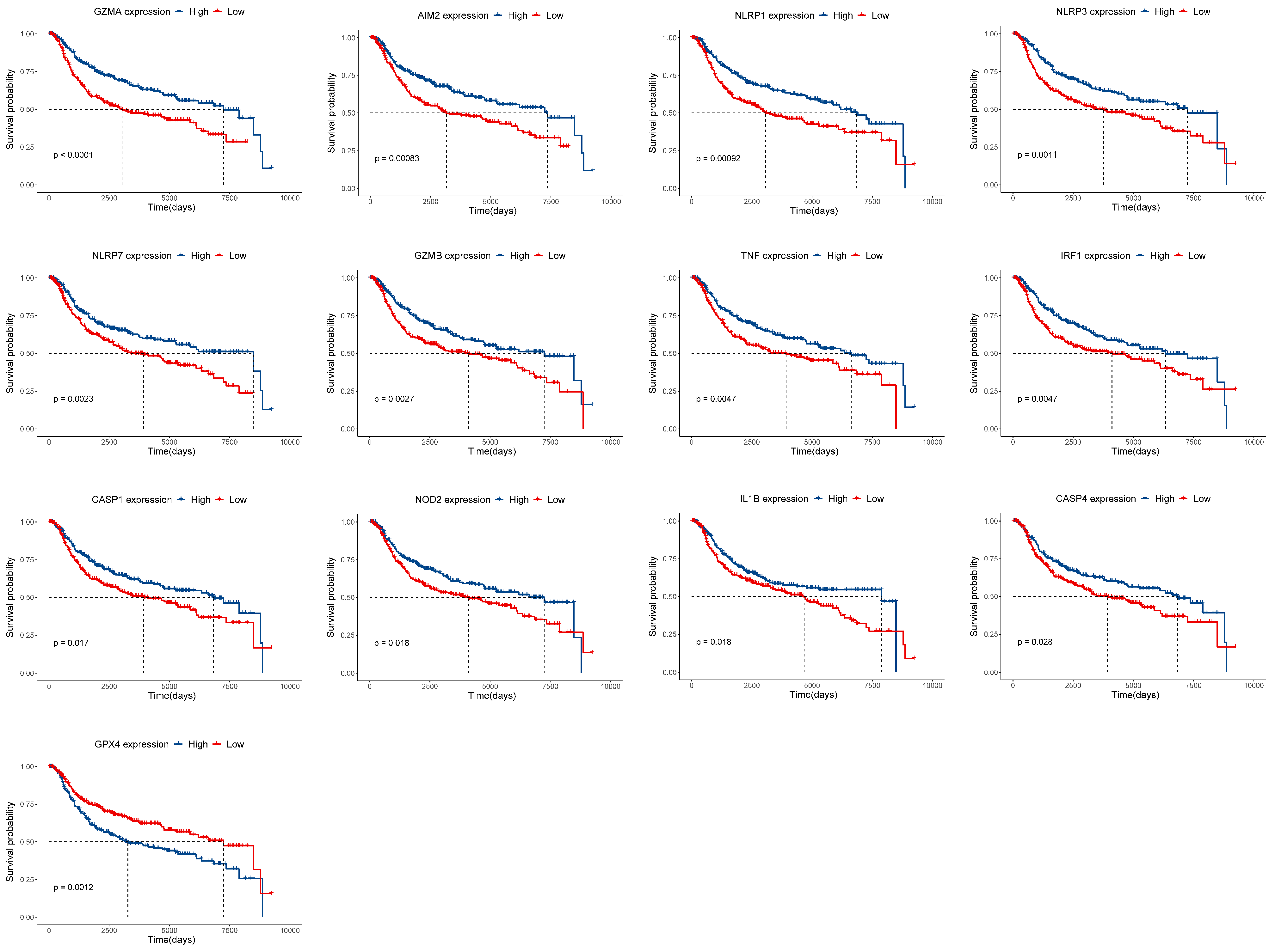


**Supplementary Figure 1.** Prognostic analysis of pyroptosis-related genes in TNBC.


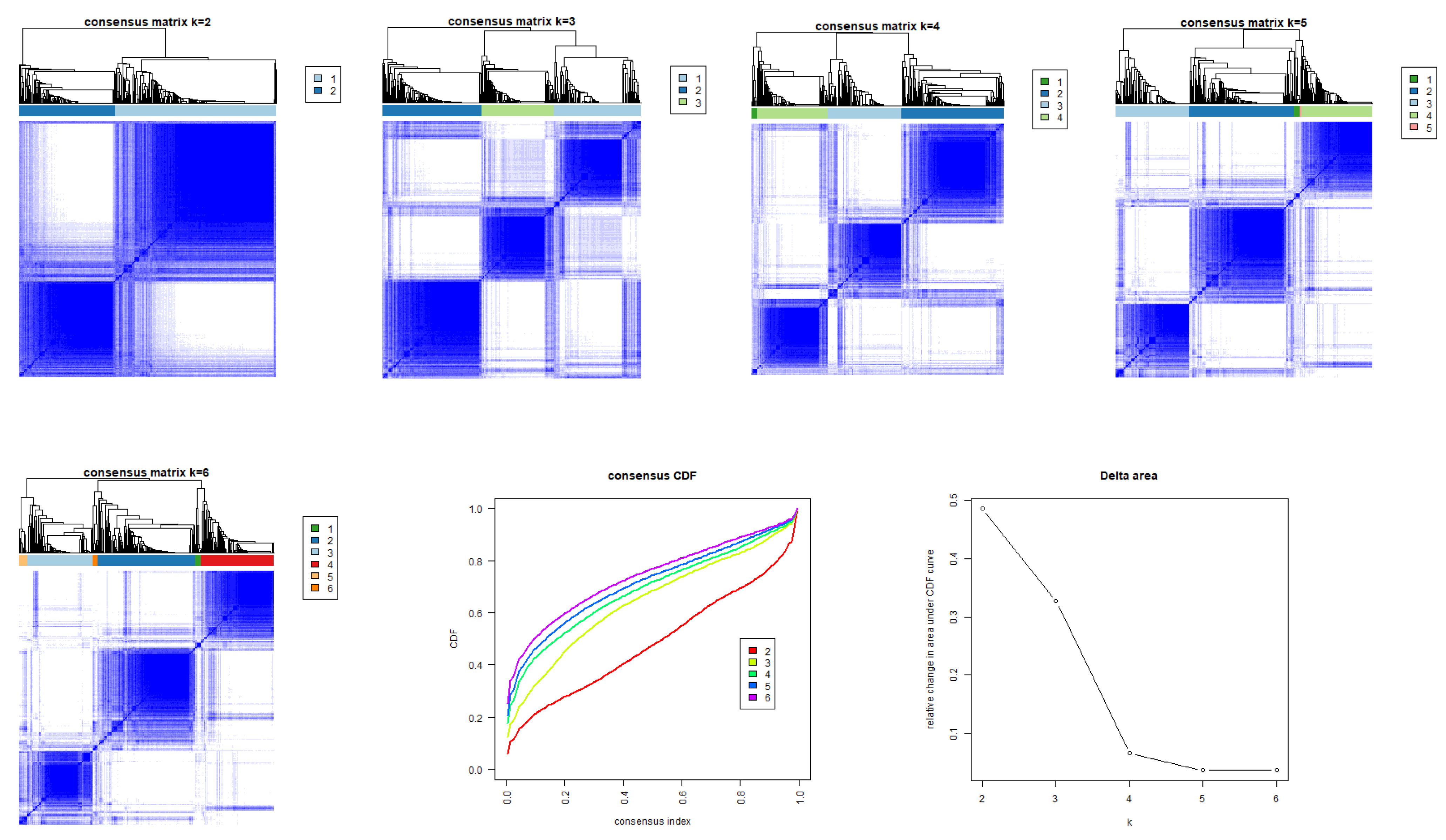


**Supplementary Figure 2.** Unsupervised clustering of pyroptosis-related genes and Consensus matrix heatmaps for k = 2-6.


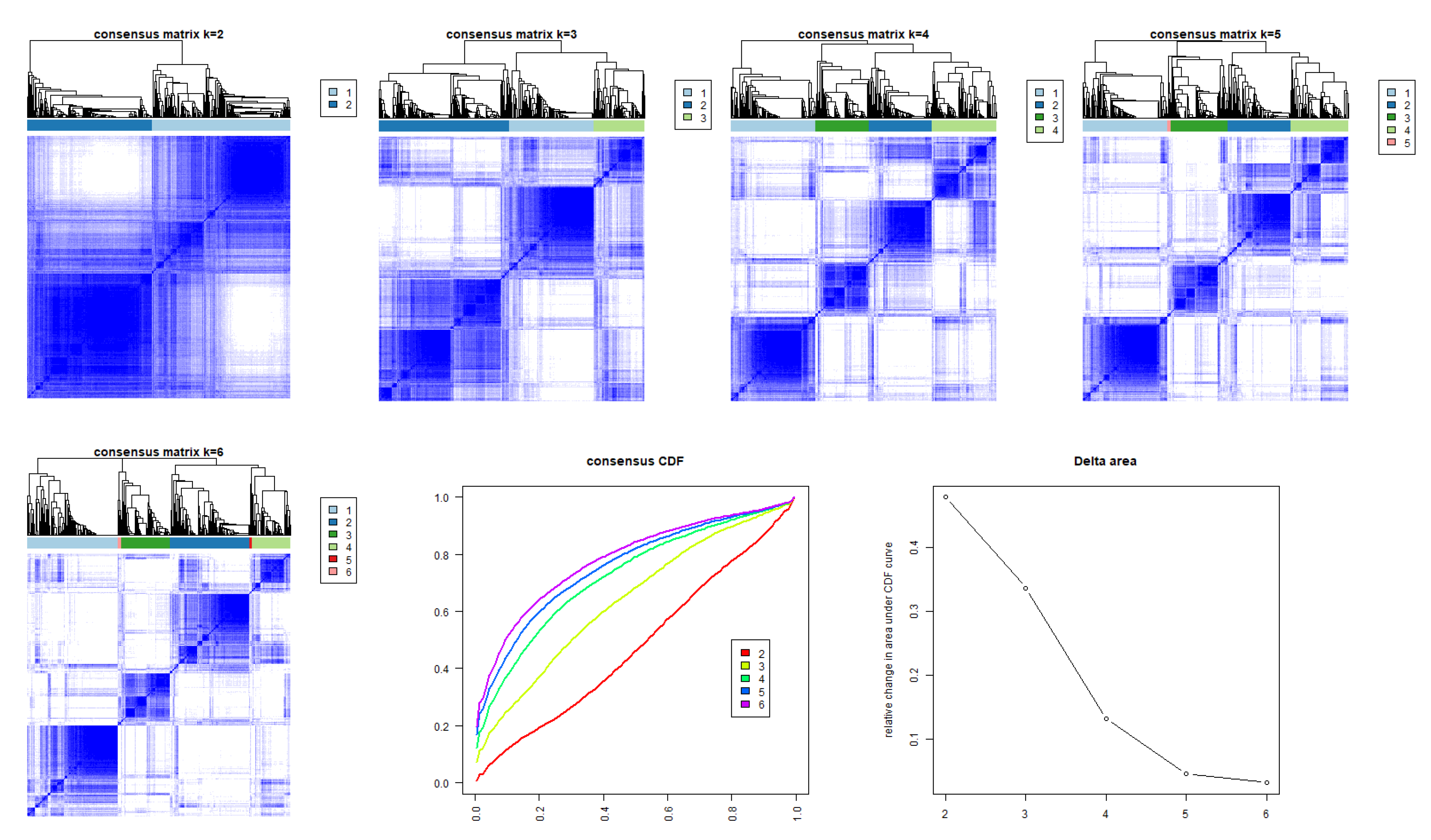


**Supplementary Figure 3.** Unsupervised clustering of DEGs and Consensus matrix heatmaps for k = 2-6.


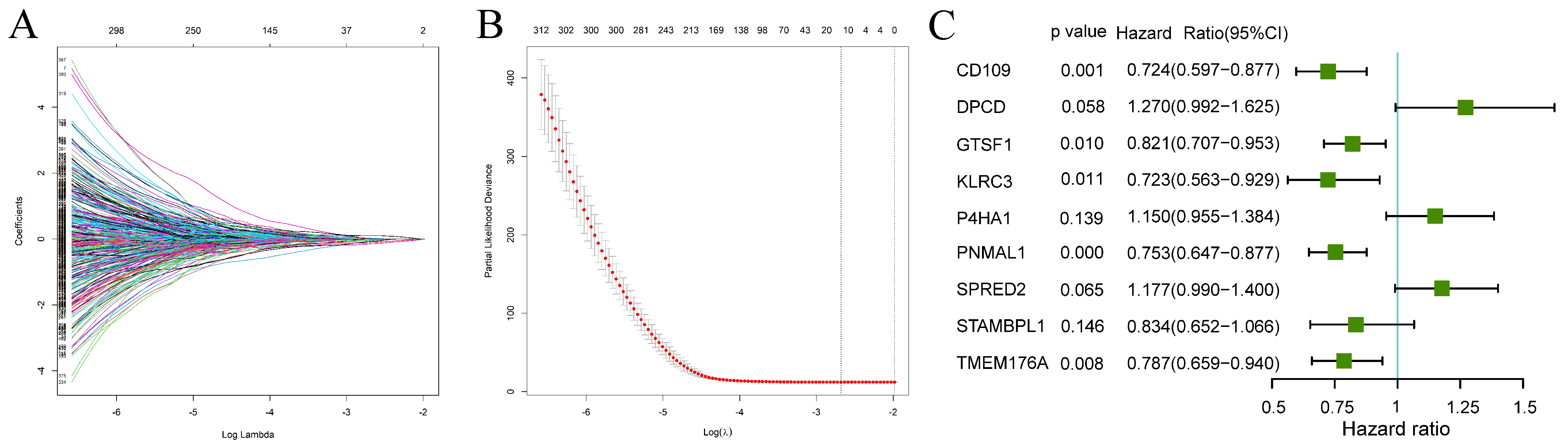


**Supplementary Figure 4.** Identifying representative candidate prognostic genes. (**A-B)** The LASSO regression analysis and partial likelihood deviance on the prognostic genes. (**C)** Forest plot of multivariate cox regression analysis for prognostic genes.


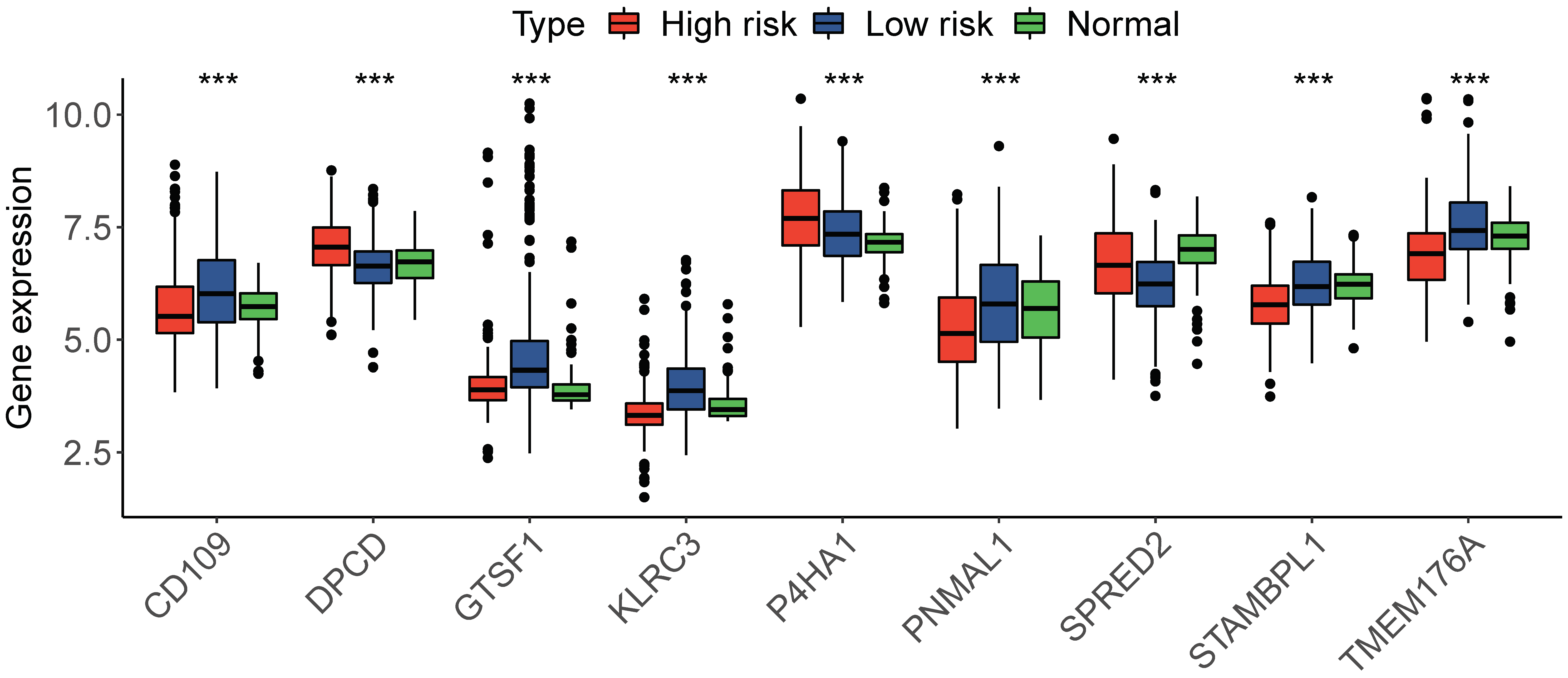


**Supplementary Figure 5.** Expression distributions of 9 genes between high-risk, low-risk and normal groups.


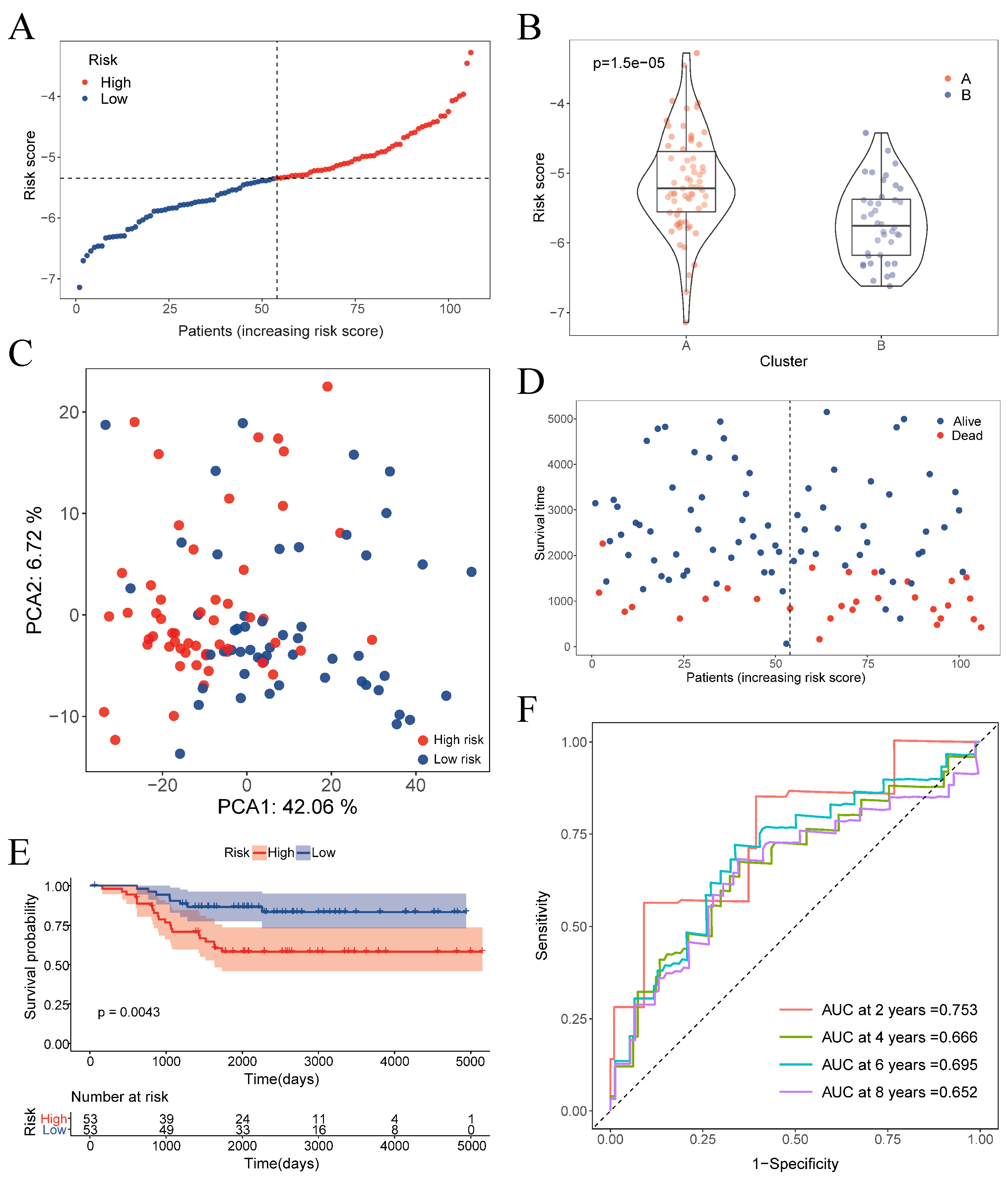


**Supplementary Figure 6.** Validation of risk score in validation set. (**A-B)** The ranked dot plot indicates the risk score distribution and scatter plot presenting the patients’ survival status. (**C)** The PCA analysis demonstrated that the patients in the different risk groups were distributed in two directions. (**D)** Differences in risk-score between pyro-clusters. (**E)** KM analysis of the OS between the two groups. (**F)** ROC curves to predict the sensitivity and specificity of 2-, 4-, 6-, and 8-year survival according to the risk-score.


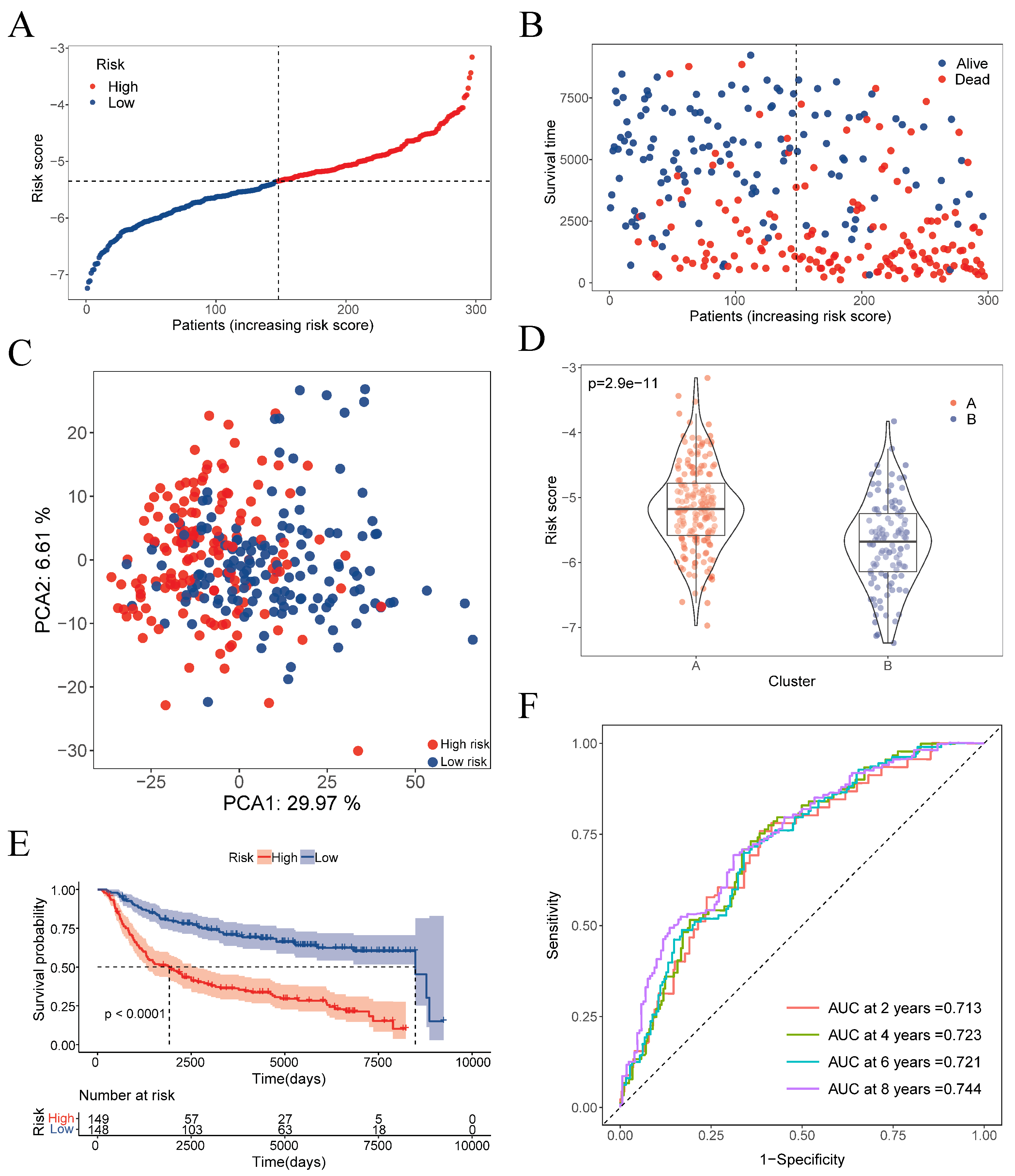


**Supplementary Figure 7.** Validation of risk score in METABRIC set. (**A-B)** The ranked dot plot indicates the risk score distribution and scatter plot presenting the patients’ survival status. (**C)** The PCA analysis demonstrated that the patients in the different risk groups were distributed in two directions. (**D)** Differences in risk-score between pyro-clusters. (**E)** KM analysis of the OS between the two groups. (**F)** ROC curves to predict the sensitivity and specificity of 2-, 4-, 6-, and 8-year survival according to the risk-score.


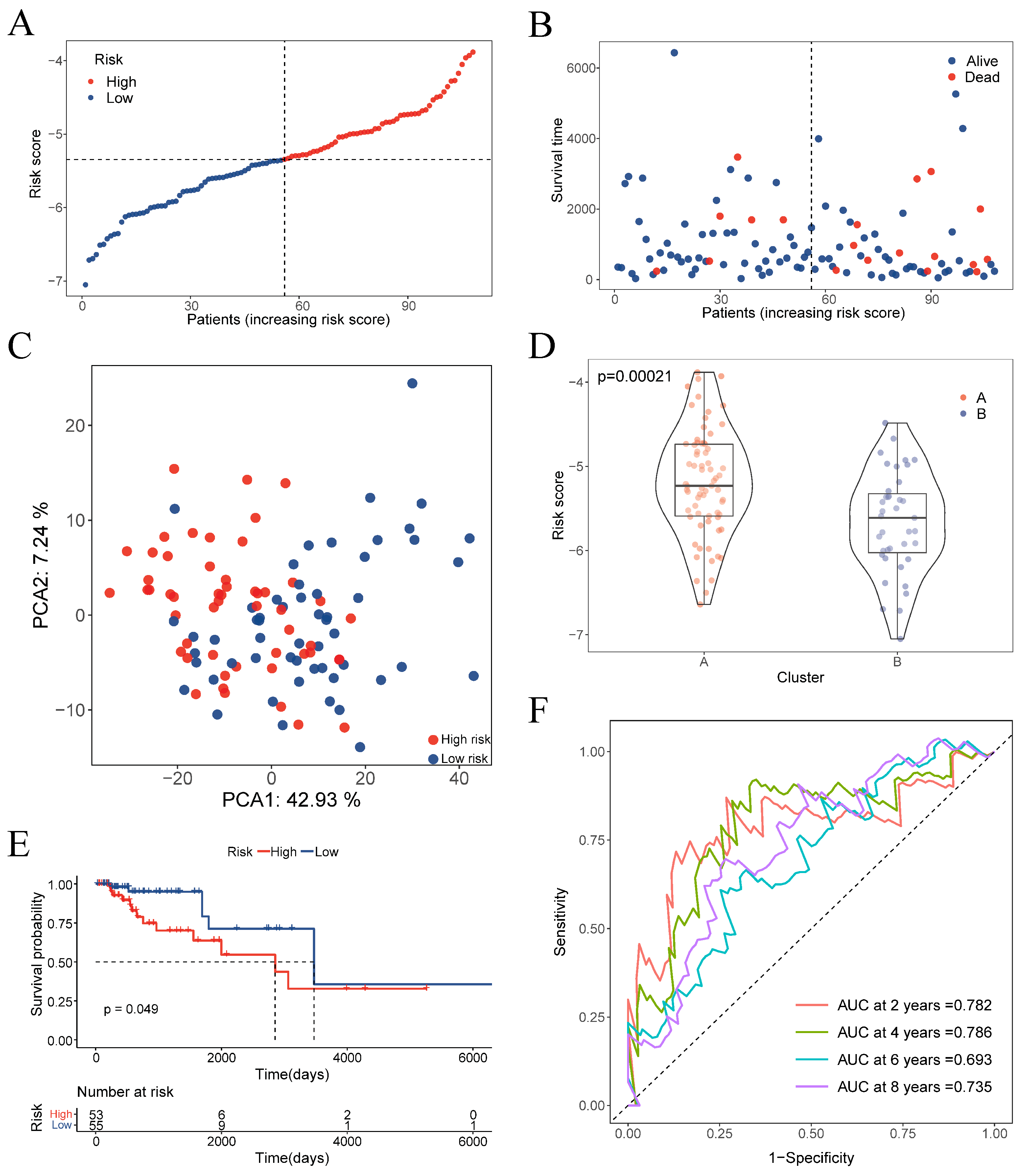


**Supplementary Figure 8.** Validation of risk score in TCGA set. (**A-B)** The ranked dot plot indicates the risk score distribution and scatter plot presenting the patients’ survival status. (**C)** The PCA analysis demonstrated that the patients in the different risk groups were distributed in two directions. (**D)** Differences in risk-score between pyro-clusters. (**E)** KM analysis of the OS between the two groups. (**F)** ROC curves to predict the sensitivity and specificity of 2-, 4-, 6-, and 8-year survival according to the risk-score.

**
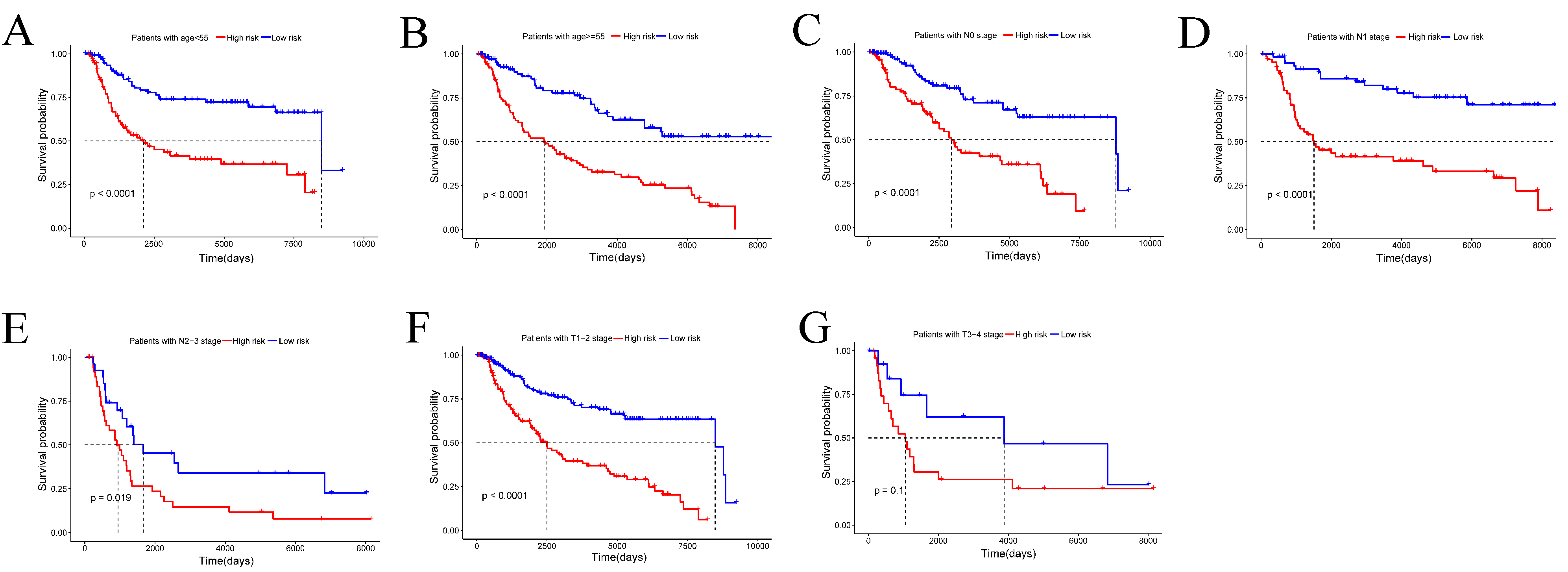
 Supplementary Figure 9.** Stratification analysis of the risk-score in TNBC. (**A-B)** Age (age<55 and age≥55 years old). (**C-E)** N stage (0, 1, and 2-3). (**F-G)** T stage (1-2 and 3-4).


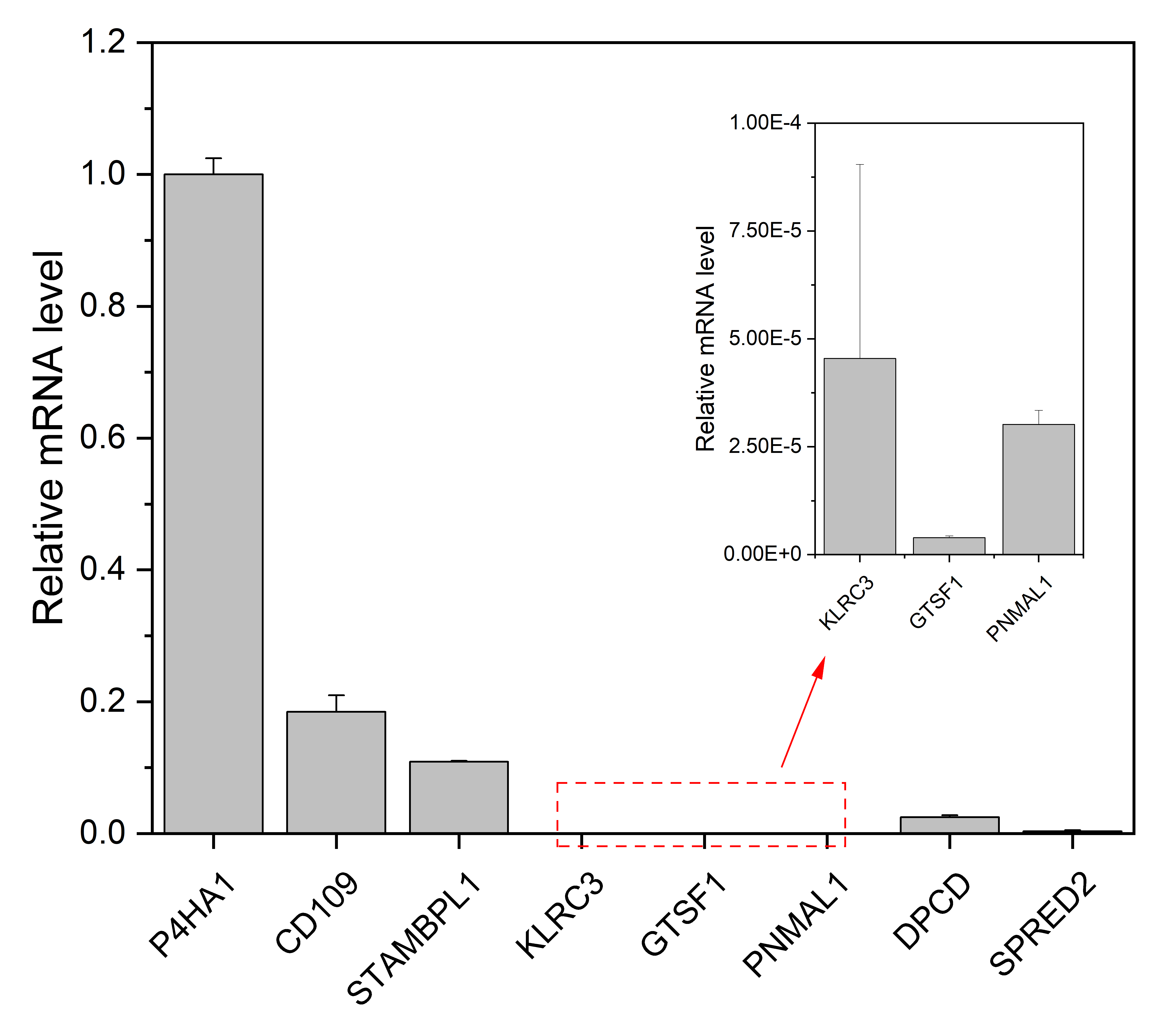


**Supplementary Figure 10.** The mRNA expression of eight genes analyzed by qRT-PCR.
